# Infective endocarditis due to *Haemophilus sputorum*

**DOI:** 10.1099/acmi.0.000410

**Published:** 2022-12-05

**Authors:** Varea H. Costello, Sara L. Robinson, Seth Klusewitz, Guy Surpris, Md Nahid, Michael G. Backlund

**Affiliations:** ^1^​ Department of Infectious Diseases, Walter Reed National Military Medical Center, Bethesda, MD, USA; ^2^​ Department of Medicine, Uniformed Services University of the Health Sciences, Bethesda, MD, USA; ^3^​ Department of Cardiology, Walter Reed National Military Medical Center, Bethesda, MD, USA; ^4^​ Department of Pathology and Laboratory Services, Walter Reed National Military Medical Center, Bethesda, MD, USA

**Keywords:** endocarditis, HACEK, *Haemophilus*, *Pasteurellaceae*

## Abstract

**Introduction.:**

*
Haemophilus* species are gram-negative, non-motile, facultative anaerobic coccobacilli in the larger family of *Pasteurellaceae *. Implicated in a variety of human diseases, *Haemophilus* species are also included in the ‘HACEK’ group of organisms, which are fastidious gram-negative bacteria, a well-described but uncommon cause of endocarditis. Among the *Haemophilus* species responsible for endocarditis, *Haemophilus parainfluenzae* is the most frequently isolated. However, novel species of *Haemophilus* have recently been described, and their clinical significance remains uncertain.

**Case presentation.:**

A 35-year-old man was admitted to the hospital after presenting with a 3 month history of nightly fevers, night sweats and unintentional weight loss, with a new murmur detected on cardiac auscultation. Blood cultures returned positive for *Haemophilus sputorum* identified by matrix assisted laser desorption ionization – time of flight MS, and confirmed with whole genome sequencing. Echocardiography revealed the presence of an aortic valve vegetation, with aortic and mitral valve leaflet perforations. He was successfully treated with surgical bioprosthetic valve replacements and pathogen-directed antibiotics without complications.

**Conclusion.:**

We describe a case of infective endocarditis due to *H. sputorum *, a newly identified *Haemophilus* species, which to the best of our knowledge has yet to be reported, and discuss the available literature regarding this organism.

## Introduction

*Haemophilus* species of bacteria are associated with a wide spectrum of pathogenicity. Several *Haemophilus* species (particularly *Haemophilus parainfluenzae* and non-encapsulated *Haemophilus influenzae *) colonize the upper respiratory tract, are commensal organisms of the oral microbiome and are unusual causes of disease [1]. By contrast, certain *Haemophilus* members can cause severe clinical presentations, ranging from bacteraemia and neonatal sepsis (encapsulated *H. influenzae *) to sexually transmitted disease (* Haemophilus ducreyi *). As the most frequently encountered organisms of *Haemophilus *, *H. influenzae* and *H. ducreyi* have traditionally been considered the most clinically relevant organisms, as infections due to other *Haemophilus* species are uncommon [2].

With improvements in bacteria identification and routine utilization of matrix-assisted laser desorption ionization – time of flight MS (MALDI-TOF MS), increasing numbers of these less common *Haemophilus* species have been isolated, which has led to a better understanding of their phenotypic characteristics and traits. Recent updates to *Haemophilus* taxonomy include the discovery of two novel species and creation of an entirely new genus within the family *Pasteurellaceae* [3]. As more of these *Haemophilus* species are isolated, further questions have arisen regarding their clinical significance.

*Haemophilus sputorum* is one these recently isolated species within the genus, first reported in 2012 by N. Norskov-Lauritsen *et al*. [4]. Apart from the isolates listed by N. Norskov-Lauritsen *et al*. in their study, to the best of our knowledge there are no published case reports concerning *H. sputorum *. We describe a case report of infective endocarditis and bacteraemia with gram-negative coccobacilli, ultimately identified as *H. sputorum *.

## Case presentation

A 35-year-old man with no remarkable medical history presented to the emergency department with a 3 month history of a dry cough, intermittent nightly fevers, a 25 pound unintentional weight loss and drenching night sweats. He reported no atypical or unexpected exposures surrounding or preceding symptom onset, specifically reporting no animal or insect bites, travel history or new sexual contacts. He also reported no family history of any malignancies.

On presentation he was tachycardic to 105, but afebrile with normal respirations and pulse oximetry. His exam was remarkable for a grade 3 holosystolic murmur audible at the apex and a grade 2/4 diastolic murmur best heard at the upper sternum. Initial laboratory results were remarkable with a white blood cell count of 5.7×10^3^ µl^−1^ (normal: 4.2–9.2×10^3^ µl^−1^) with a mild neutrophilia of 73.2 % (normal: 43.0–68.8 %), an erythrocyte sedimentation rate >120 mm h^−1^ (normal: <30 mm h^−1^) and a C-reactive protein level of 9.06 mg dl^−1^ (normal: 0.00–0.49 mg dl^−1^).

**Fig. 1.**
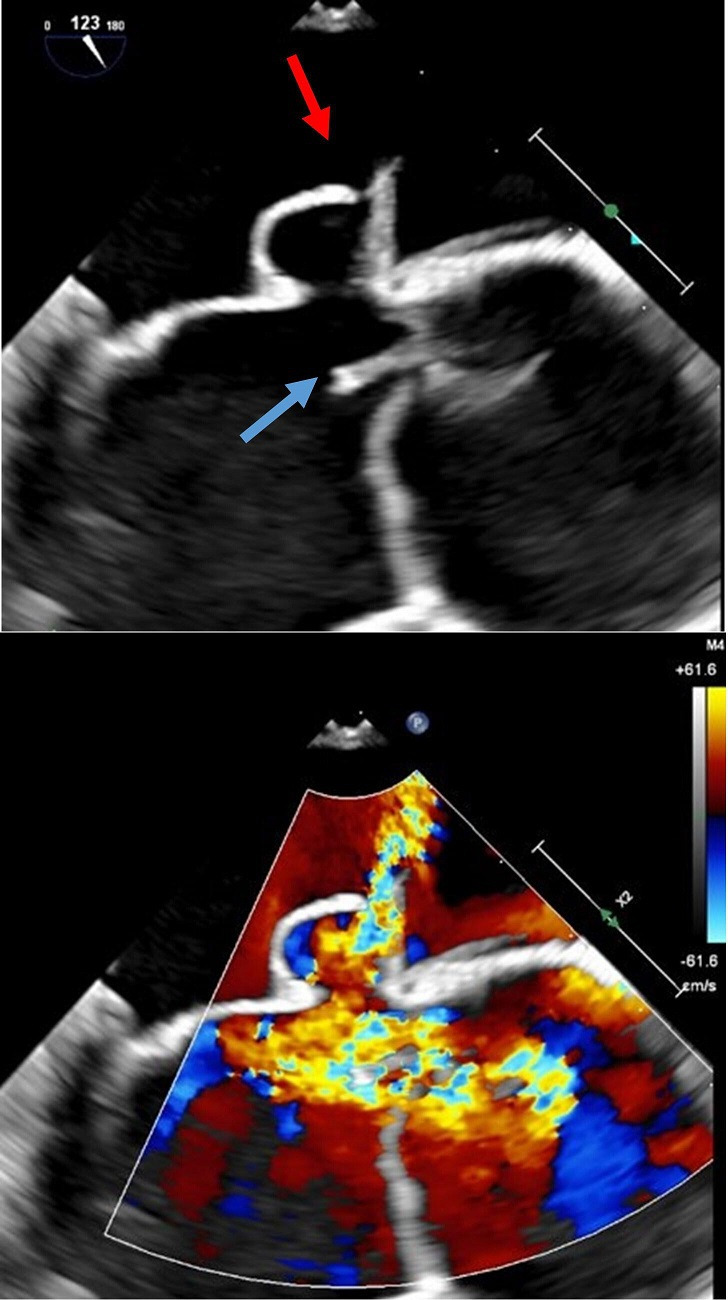
Top: transoesophageal echocardiogram demonstrating valvular vegetation (blue arrow) and mitral valve outpouching and perforation (red arrow). Bottom: the regurgitant jet through the aortic valve perforation (courtesy of Walter Reed Cardiology).

A transthoracic echocardiogram was first obtained, which revealed a normal left ventricular ejection fraction, a previously unknown bicuspid aortic valve and probable mitral valve vegetation. A transoesophageal echocardiogram was subsequently performed, which demonstrated severe aortic valve regurgitation with a perforation of the non-coronary cusp associated with an 8 mm, thin, filamentous mobile structure consistent with a vegetation (Fig. 1). Furthermore, the anterior mitral valve leaflet was perforated at the location of the intervalvular fibrosa with evidence of moderate regurgitation through this defect. A brain magnetic resonance image was obtained to assess for any embolic phenomenon, and was remarkable for bilateral cerebral and cerebellar microbleeds consistent with probable embolic complications from endocarditis, with evidence of active oedema and enhancement of the lesions in the left frontoparietal lobe.

Upon admission, he was empirically started on treatment for endocarditis with 2 g of ceftriaxone every 24 h and 1 g of vancomycin every 12 h. Three sets of blood cultures were drawn prior to his first dose of antibiotics, and all aerobic bottles (total of three) were positive for *Haemophilus* species within 72 h. Conventional multiplex PCR was performed with the BioFire Blood Culture Identification panel run on the BioFire Torch Platform (Biofire), and was positive for *H. influenzae *. Further testing with MALDI – TOF MS [BD Bruker MALDI Biotyper, version 3.2, CA library version 10 (claim 6)], from single colonies that were sub-cultured from chocolate agar plates (Remel, Cat#R01302), identified *H. sputorum *. Whole genome sequencing (MiSeq benchtop sequencer, reagent kit v3) revealed this isolate demonstrated >95 % intra-species sequence identity with *H. sputorum,* confirming identification. Antimicrobial susceptibility testing demonstrated susceptibility to ampicillin, ceftriaxone, levofloxacin and trimethoprim-sulfamethoxazole. The isolate was submitted to the NCBI GenBank, with the accession number SAMN29768393.

Based on these results, the patient was then narrowed to monotherapy with ceftriaxone, and approximately 2 weeks later, he successfully underwent surgical bioprosthetic aortic and mitral valve replacements with aorto-mitral curtain reconstruction, with no intra- or postoperative complications. Four weeks after his valve replacement, he had a complication with his peripherally inserted central catheter line, requiring removal, and started on oral levofloxacin and completed a total 6 week course of antibiotics after valve replacement without any further complications. He continued to remain clinically well at his follow-up appointments, with a postoperative repeat echocardiogram showing a normal ejection fraction and appropriately functioning mechanical valves.

## Discussion

The genus *Haemophilus* is classified within the larger family *Pasteurellaceae *, and comprises facultatively anaerobic, gram-negative pleomorphic rods characterized by their fastidious growth requirements. With improvements in bacterial isolation and identification, organisms of the family *Pasteurellaceae* have undergone several recent revisions, including discovery of novel species (* Haemophilus pittmaniae* and *H. sputorum *) and creation of a new genus (* Aggregatibacter *). *Haemophilus* remains the most clinically relevant pathogen among this family, with several members causing well-known clinical entities [1].

*Haemophilus* encompasses a total of nine distinct species with disease-causing potential. Unique for their fastidious growth requirements, *Haemophilus* requires media supplementation with the stimulating factors of X factor (haemin) and/or V factor (NAD) for growth.

The nine *Haemophilus* species are classified into three groups based on phenotypic characteristics: *H. influenzae, H. aegyptius* or *H. ducreyi* groups [5]. Variability in requirements of these growth factors, in conjunction with other distinguishing characteristics (such as expression of catalase or the ability to cause haemolysis), allows for differentiation among the species.

**Fig. 2.**
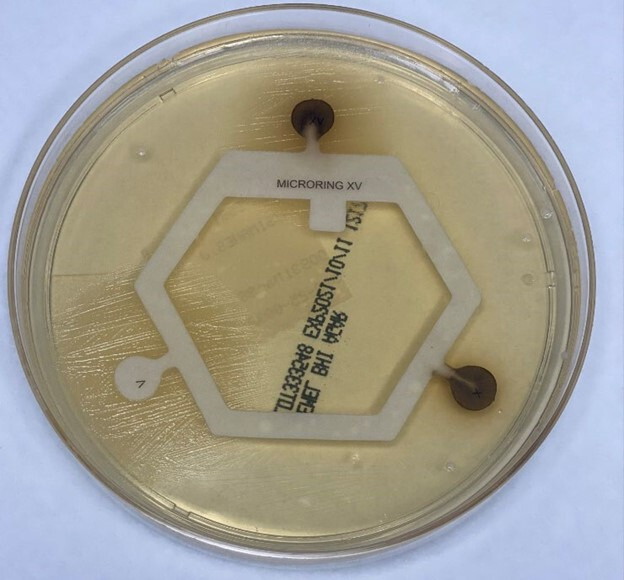
*
H. sputorum* isolate showing growth in the presence of V and XV factors, but not X factor, on a brain-heart infusion agar plate.

*H. sputorum* was first described in 2012, when it was incidentally discovered by Norskov Lauritsen *et al*. during an experiment attempting to investigate the difference between two other *Haemophilus* species [4]. Similar to *H. parainfluenzae *, *H. sputorum* requires V factor, but not X factor, to grow (Fig. 2). Recent studies have also demonstrated that *H. sputorum* possesses a complete capsule locus transcribed *in vitro*, with high resemblance to the capsule of *H. influenzae *, an unusual finding: capsulation has otherwise only been identified in *H. influenzae* among the family *Pasteurellaceae* with host specificity for humans [6]. In fact, *H. sputorum* has cross-reacted with standard PCR testing targeting the capsular *bexA* gene for *H. influenzae *, with speculation that this gene may have been acquired by *H. sputorum* via horizontal gene transfer [6].

The clinical characteristics of *H. sputorum* are ill defined. To date, there do not appear to be any published case reports with *H. sputorum* associated with an infectious process. In the study by Norskov-Lauritsen *et al*. first describing *H. sputorum *, nine isolates were recorded: only one of the isolates was from blood, and several were from throat and sputum samples. These samples were obtained over a nearly 30 year period, from 1983 to 2009 [4]. There have been no documented cases of *H. sputorum* and endocarditis, though there is evidence demonstrating that *Haemophilus* species (particularly *H. parainfluenzae *) are an increasingly recognized cause of endocarditis [7, 8]. This lack of data may be related to the limitations of bacterial isolation, or perhaps simply like other members of the genus *Haemophilus *, the pathogenicity and clinical impact of *H. sputorum* remain low. *H. sputorum* and the clinical implications should be better elucidated in the upcoming years, as reference databases and libraries are updated in conjunction with more widespread use of MALDI-TOF [5].

Given the lack of available of data on *H. sputorum *, currently there are no particular considerations regarding treatment. As with any other infection due to *Haemophilus* species, treatment should be guided by antimicrobial susceptibility results. Susceptibility patterns of all *Haemophilus* species are comparable to those of *H. influenzae *, and as an increasing amount of *H. influenzae* species produce beta-lactamases (∼30 % of strains are resistant to penicillins), penicillins should be avoided until susceptibility results return [9]. Third-generation cephalosporins, fluoroquinolones, trimethoprim/sulfamethoxazole and aztreonam typically have good activity. Indeed, the treatment recommendations from the American Heart Association for native valve endocarditis due to HACEK group is 4 weeks with either a third-generation cephalosporin or a fluoroquinolone, given the increasing detection of beta-lactamases within these organisms [9]. In our case, the patient successfully underwent treatment with valvular surgery followed by an extended course of intravenous and highly bioavailable oral antibiotics with no complications.

## Conclusion

In this report, we present a case of *H. sputorum* bacteraemia leading to acute infective endocarditis of the aortic and mitral valves, complicated by embolic sites of infection. Closely related to *H. parainfluenzae *, *H. sputorum* requires V factor but not X factor for growth, but also harbours a complete capsule locus, similar to *H. influenzae *. To the best of our knowledge, this is the first published case of *H. sputorum* infective endocarditis and second report of *H. sputorum* isolated from blood. More information regarding *H. sputorum* is necessary to appreciate the prevalence and clinical significance of this organism.
